# An extremely rare primary sarcoma of the lung with peritoneal and small bowel metastases: a case report

**DOI:** 10.1186/s12957-019-1691-8

**Published:** 2019-08-19

**Authors:** Sanja Pleština, Nikša Librenjak, Ante Marušić, Lovorka Batelja Vuletić, Zoran Janevski, Marko Jakopović

**Affiliations:** 1 Department of Respiratory Diseases, UHC Zagreb, University of Rijeka School of Medicine, Jordanovac 104, 10000 Zagreb, Croatia; 2 Department of Oncology, UHC Zagreb, Kišpatićeva 12, 10000 Zagreb, Croatia; 3 Department of Radiology, UHC Zagreb, Kišpatićeva 12, 10000 Zagreb, Croatia; 4 Department of Pathology, UHC Zagreb, University of Zagreb School of Medicine, Kišpatićeva 12, 10000 Zagreb, Croatia; 5 Department of Surgery, UHC Zagreb, Jordanovac 104, 10000 Zagreb, Croatia; 6 Department of Respiratory Diseases, UHC Zagreb, University of Zagreb School of Medicine, Jordanovac 104, 10000 Zagreb, Croatia

**Keywords:** Primary pulmonary sarcoma, Undifferentiated pleomorphic sarcoma, Intestinal metastasis, Lung neoplasms

## Abstract

**Background:**

Primary sarcoma of the lung is a very rare malignant tumor accounting for less than 0.5% of all lung tumors and presenting diagnostic and treatment challenge. We describe a case of a patient diagnosed with primary lung undifferentiated pleomorphic sarcoma developing subsequent peritoneal and small bowel metastases, which stand for highly unusual disease presentation.

**Case presentation:**

A 57-year-old male presented with extensive partially necrotic tumor in the left upper lobe (LUL) of the lung that involved LUL bronchus and extended to the visceral pleura. There was no evidence of nodal or visceral dissemination. After initial presentation, the patient was admitted to the hospital’s pulmonology department for further workup. The most likely diagnosis based on biopsy specimen was poorly differentiated sarcoma. Left pneumonectomy with mediastinal lymph node dissection was performed. The final pathohistological diagnosis (PHD) was undifferentiated pleomorphic sarcoma (UPS). Three months after lung surgery, a follow-up CT scan was done which showed a 60-mm obstructive metastatic intraabdominal lesion with small bowel infiltration and further separate peritoneal deposits. Unfortunately, an urgent surgery had to be performed as the patient developed signs of acute abdomen due to bowel perforation. Only 2 months later, the patient passed away at home.

**Conclusions:**

Treatment options of UPS are based on algorithms used in treatment of extremity lesions with well-established role of surgery. However, the role of perioperative chemotherapy remains equivocal with no strong evidence-based data due to the rarity of the disease. Small bowel is an unexpected metastatic site, but of significant clinical relevance.

## Background

The undifferentiated pleomorphic sarcoma (UPS) is a rare tumor accounting for less than 0.2% of all lung tumors [1–3] and therefore presents diagnostic and treatment challenge. This entity was named malignant fibrous histiocytoma until 2012 when it was reclassified as undifferentiated pleomorphic sarcoma by the World Health Organization. Soft tissue sarcoma usually affects extremities or abdomen and pelvis, although it can occur anywhere in the body [4]. Lung metastases from extrapulmonary primary sarcomas have been more frequently reported than primary pulmonary sarcomas. Since the first reported case of primary pulmonary MFH 40 years ago, there have been approximately 50 additional cases reported in literature available in English [3, 5]. In this paper, we present the case of a patient with primary lung undifferentiated pleomorphic sarcoma with subsequent intestinal and peritoneal metastases which is quite unusual presentation of disease.

## Case presentation

**Fig. 1:**
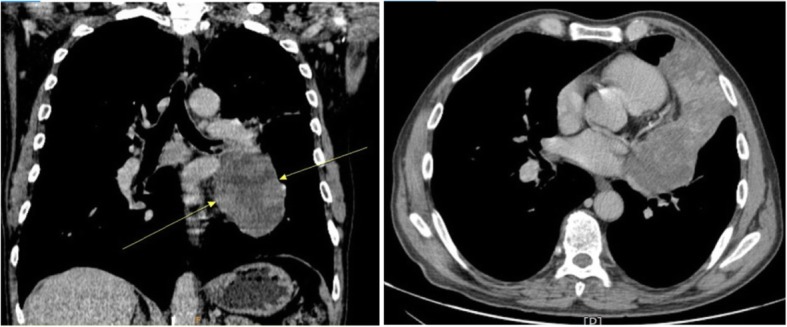
An extensive partially necrotic tumor in the LUL of the lung

A 57-year-old male was admitted to the Pulmonary Oncology Department of University Hospital Centre Zagreb in November 2017 with cough and hemoptysis. He smoked 27 packs/year, had an alcohol abuse history, and displayed no evidence of present or past soft tissue neoplasms or history of radiation exposure. His Karnofsky performance score was 80. Subsequent physical examination revealed reduced air entry in the left lung. His complete blood count and biochemical parameters were within the normal range. Initial chest x-ray showed an extensive consolidation in the left lung suggesting possible malignancy; CT of chest and abdomen was recommended. The CT scan showed an extensive partially necrotic tumor in the left upper lobe (LUL) extending from the left hilum with infiltration of the LUL bronchus to the visceral pleura, measuring 74 mm in the largest diameter (Fig. 1).

There was no evidence of nodal or visceral dissemination at presentation. Bronchoscopy examination with biopsy was performed, and specimen from the LUL bronchus was taken. Histopathological analysis showed an almost completely necrotic tumor with a share of less than 10% viable pleomorphic tumor cells expressing vimentin and CD99 and focal positive desmin. There was no expression of AE1/AE3, p40, TTF-1, napsin A, epithelial membrane antigen (EMA), CK7, and S100. The most likely diagnosis was poorly differentiated sarcoma.

**Fig. 2:**
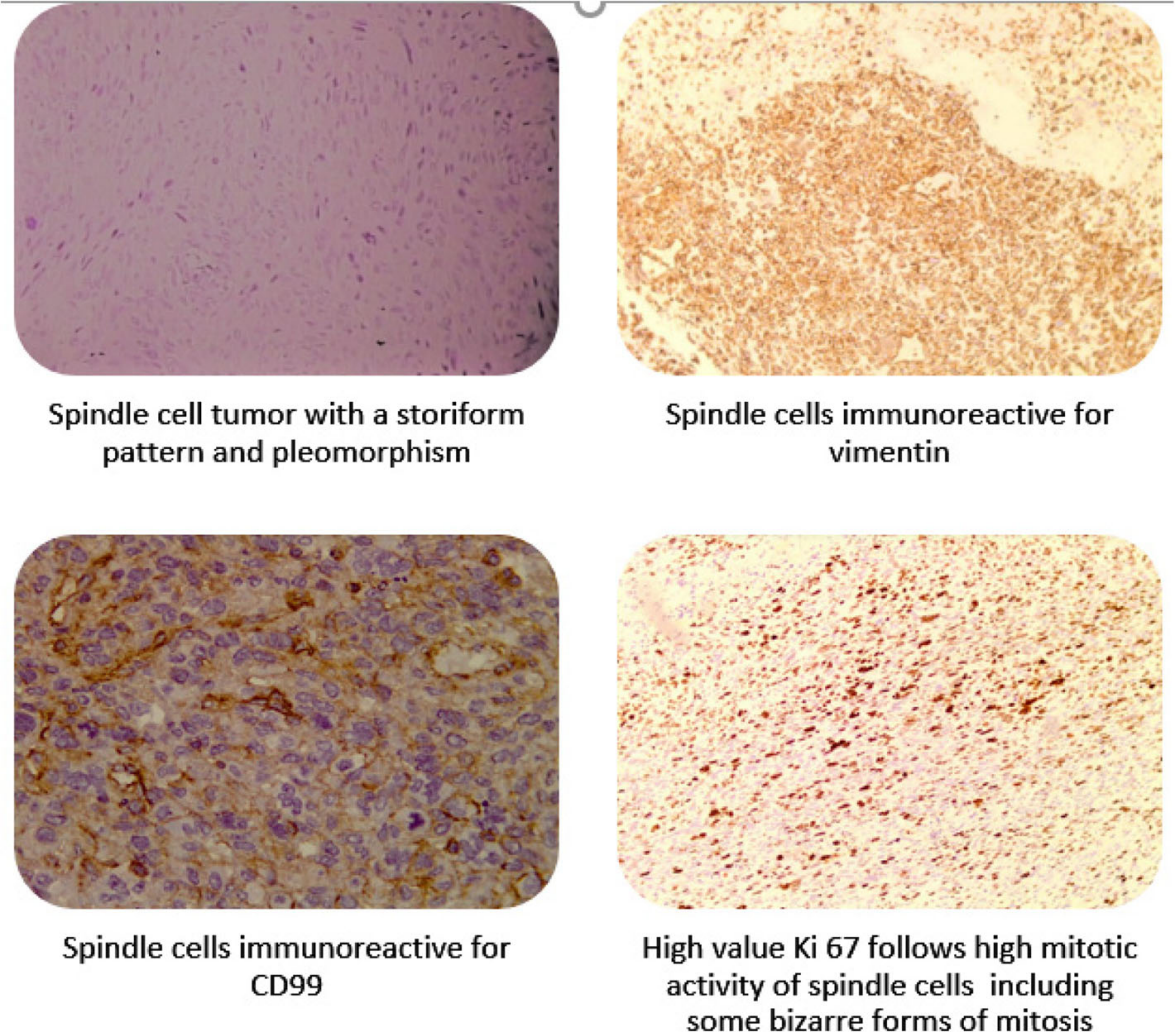
Immunohistochemistry and microscopic characteristics of UPS

For final staging, PET/CT scan was performed, showing no extra thoracic spread of the disease. The multidisciplinary tumor board deemed that lung surgery was the best treatment alternative, and several weeks later, left pneumonectomy with mediastinal lymph node dissection was done. Final pathohistological analysis noted a tumor size of 100 × 85 × 71 mm, more than 80% of which was necrotic. The pleomorphic tumor cells showed the same immunohistochemical profile as in biopsy specimen. Synovial sarcoma-associated translocation was negative. All surgical margins and dissected mediastinal lymph nodes were tumor free. Based on this, the final diagnosis of undifferentiated pleomorphic sarcoma (grade 3) was made (Fig. 2).

**Fig. 3:**
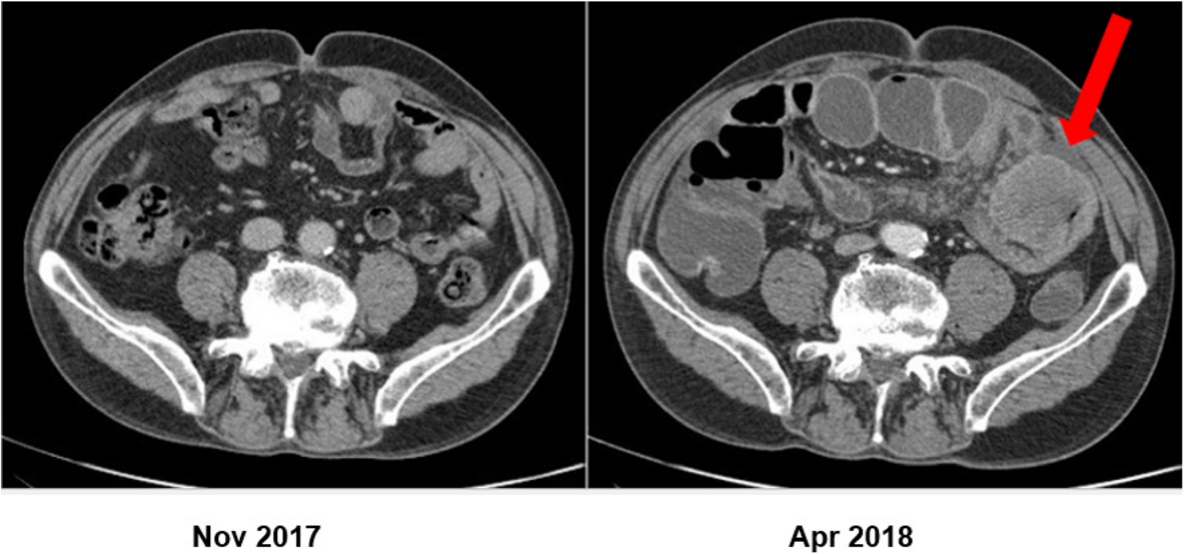
Comparative CT scan of the abdomen in November 2017 and April 2018 with new developed intraabdominal metastasis and small bowel dilatation

The patient recovered well from the surgery, and follow-up was planned. At the first follow-up visit 7 weeks after lung surgery, the patient was free of malignant disease as per physical examination and chest x-ray. Three months after the lung surgery, a follow-up CT scan was done which showed a 60-mm metastatic intraabdominal lesion with an evident small bowel involvement, as well as further separate malignant peritoneal deposits. The scan also showed mild upstream dilatation of the small bowel suggestive of partial obstruction (Fig. 3).

**Fig. 4:**
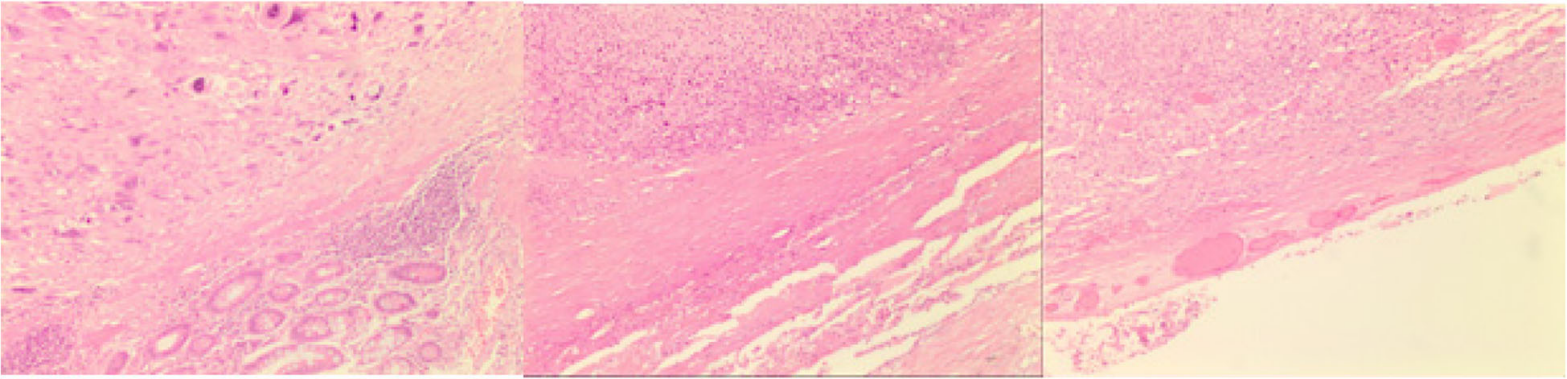
Histological slides of the lung lesion and of the small bowel lesion

Therefore, an abdominal surgery was planned. However, just a couple of days later, the patient presented to the Emergency Department with clinical signs of acute abdomen due to bowel perforation. Urgent abdominal surgery was performed with resection of perforated segment of the ileum, which was infiltrated with tumor. A unipolar ileostomy was then fashioned. The histopathological analysis confirmed metastasis of undifferentiated sarcoma (Fig. 4).

The patient was discharged from the hospital 9 days later with the Karnofsky performance score of 40; thus, the best supportive care was indicated. The patient died at home 2 months later.

## Discussion

Primary pulmonary sarcomas are extremely rare, and can be found in 0.1 to 0.5% of all pulmonary neoplasm cases. The most common sarcomas include leiomyosarcoma, malignant fibrous histiocytoma, and synovial sarcoma [6, 7]. Symptoms and radiologic appearances are similar to those of more common lung carcinomas. The radiological features of the sarcomas are variable, not lesion-specific, and not sufficient to suggest specific diagnosis [8]. Presentation of the disease depends more on tumor localization than histopathological features [9]. After epithelial malignancy is ruled out, the most important differential diagnosis of primary pulmonary sarcoma is metastatic spread from an extrapulmonary sarcoma. Other differential diagnoses include pleomorphic lung carcinoma, pulmonary sarcomatoid carcinoma, and malignant melanoma. Therefore, a detailed medical history and appropriate diagnostic examination is necessary to specify that the tumor has primary pulmonary origin. As patients diagnosed with pulmonary sarcoma are quite rare, it is difficult to define a reliable management protocol for such patients [2].

Immunohistochemistry has an important role for accurate diagnosis and classification of sarcoma type. It is often positive for keratin, actin, desmin, EMA, CD99, and CD34, but positive stains usually do not help diagnosis, so UPS is a diagnosis of exclusion [10]. In our case, there was no evidence of epithelial differentiation. The S-100 protein, which can be found positive in malignant peripheral nerve sheath tumor and melanoma, was negative. Synovial sarcoma is characterized by mast cells within the tumor, but none were detected in this case. Characteristic translocation for synovial sarcoma was negative. Microscopic description characteristic for UPS was also present in the tumor tissue of our patient: storiform pattern, irregular fascicles, variable cellularity, and pleomorphic and bizarre tumor cells with foamy cytoplasm and marked atypia; on the background of inflamed collagenous stroma, multinucleated giant cells were seen, also numerous mitotic figures, including atypical forms. Grading of tumor was determined as per FNCLCC system criteria.

Treatment options are based on algorithms used in treatment of extremity lesions with the well-established role of wide surgical resection aiming for tumor-free margins as the primary therapeutic modality. Some authors found that completeness of resection was correlated with significantly increased survival, but size and grade of tumor were not [11]. Radical resection is established as the only treatment that can achieve cure or prolonged survival if the tumor seems resectable [2, 12, 13]. On the other hand, the role of perioperative chemotherapy is still controversial [13, 14]. According to recent data, perioperative chemotherapy does not improve overall survival. The only independent factor associated with better survival is curative resection with a microscopically negative margin [15]. There is evidence of higher rate of nodal involvement in primary pulmonary sarcomas than in case of extremity soft tissue sarcomas. Therefore, the patients with a primary pulmonary sarcoma have a markedly worse prognosis [16]. Considering this evidence, we decided not to treat the patient with neoadjuvant chemotherapy. After upfront radical surgery with extensive mediastinal lymphadenectomy, which showed clear margins and no nodal involvement, we decided not to give adjuvant chemotherapy either. A close follow-up was advised due to relatively high recurrence rates.

The role of adjuvant radiation therapy still remains equivocal. It can provide acceptable local control of some soft tissue sarcomas after surgical resection [17], but there are also published data not in favor of adjuvant radiotherapy [2]. Therefore, there are no strong evidence-based data for radiation therapy to be an integral part of adjuvant treatment of the primary lung sarcoma.

These neoplasms have an aggressive clinical course with a high potential for recurrence and metastasis. In an advanced stage, a combination of chemotherapy or radiotherapy may be used as a palliative approach, although the tumor seems insensitive to both chemotherapy and radiotherapy [3]. The overall median survival is 24 to 48 months according to retrospective reviews of primary pulmonary sarcoma patients with different histological types (mOS 17 months for grade 3 sarcomas) [9, 12]. However, despite the aggressive behavior of pulmonary sarcoma, there are several reports of patients with long-term survival.

During the short follow-up period, our patient developed unexpected peritoneal dissemination, which, to the best of our knowledge, has not been reported as a site of metastatic spread for primary pulmonary sarcoma yet. There has been one case report of cardiac metastasis from an undifferentiated pleomorphic sarcoma of the lung presenting with symptomatic right heart failure described in the literature [18], and treated with surgical resection.

High-risk features in our case were poorly differentiated histology and tumor size; thus, despite radical surgery with completeness of resection, clear margins, and no nodal involvement, the patient passed away in less than a year after diagnosis was made.

## Conclusions

Primary undifferentiated pleomorphic lung sarcoma remains an extremely rare malignancy without standardized treatment and poor prognosis. An optimal treatment strategy has not yet been elucidated due to limited data available, although complete surgical excision remains the preferable treatment option. Further investigation and data collection from clinical practice are needed to improve the outcomes, optimize treatments, and define the follow-up approach to this aggressive malignancy.

## Data Availability

The data are available from the corresponding author on reasonable request.

## Abbreviations

CT: Computerized tomography
EMA: Epithelial membrane antigen
FNCLCC: Fédération Nationale des Centres de Lutte Contre Le Cancer
LUL: Left upper lobe
MFH: Malignant fibrous histiocytoma
PET/CT: Positron emission tomography/computed tomography
PHD: Pathohistological diagnosis
UPS: Undifferentiated pleomorphic sarcoma
